# The Effects of Novel Co-Amorphous Naringenin and Fisetin Compounds on a Diet-Induced Obesity Murine Model

**DOI:** 10.3390/nu16244425

**Published:** 2024-12-23

**Authors:** Sarai Vásquez-Reyes, Miranda Bernal-Gámez, Jorge Domínguez-Chávez, Karina Mondragón-Vásquez, Mónica Sánchez-Tapia, Guillermo Ordaz, Omar Granados-Portillo, Diana Coutiño-Hernández, Paulina Barrera-Gómez, Nimbe Torres, Armando R. Tovar

**Affiliations:** 1 Departamento de Fisiología de la Nutrición, Instituto Nacional de Ciencias Médicas y Nutrición Salvador Zubirán, CDMX, México 14080, Mexico; lnsaraivreyes@gmail.com (S.V.-R.); mirbergam@gmail.com (M.B.-G.); monica.sanchezt@incmnsz.mx (M.S.-T.); guillermo.ordazn@incmnsz.mx (G.O.); omar.granadosp@incmnsz.mx (O.G.-P.); dianacristalch@gmail.com (D.C.-H.); pauli.bag13@gmail.com (P.B.-G.); nimbe.torrest@incmnsz.mx (N.T.); 2 Facultad de Bioanálisis Región Veracruz, Universidad Veracruzana, Agustín de Iturbide, Veracruz 91700, Mexico; jorgedominguez@uv.mx (J.D.-C.); kmondragon@uv.mx (K.M.-V.)

**Keywords:** co-amorphous, naringenin, fisetin, energy expenditure, glucose tolerance

## Abstract

Background/Objective: In recent studies, it has been shown that dietary bioactive compounds can produce health benefits; however, it is not known whether an improvement in solubility can enhance their biological effects. Thus, the aim of this work was to study whether co-amorphous (CoA) naringenin or fisetin with enhanced solubility modify glucose and lipid metabolism, thermogenic capacity and gut microbiota in mice fed a high-fat, high-sucrose (HFSD) diet. Methods: Mice were fed with an HFSD with or without CoA-naringenin or CoA-fisetin for 3 months. Body weight, food intake, body composition, glucose tolerance, hepatic lipid composition and gut microbiota were assessed. Results: CoA-naringenin demonstrated significant reductions in fat-mass gain, improved cholesterol metabolism, and enhanced glucose tolerance. Mice treated with CoA-naringenin gained 45% less fat mass and exhibited improved hepatic lipid profiles, with significant reductions seen in liver triglycerides and cholesterol. Additionally, both CoA-flavonoids increased oxygen consumption (VO_2_), contributing to enhanced energy expenditure and improved metabolic flexibility. Thermogenic activation, indicated by increased UCP1 and PGC-1α levels, was observed with CoA-fisetin, supporting its role in fat oxidation and adipocyte size reduction. Further, both CoA-flavonoids modulated gut microbiota, restoring diversity and promoting beneficial bacteria, such as *Akkermansia muciniphila*, which has been linked to improved metabolic health. Conclusions: These findings suggest that co-amorphous naringenin or fisetin offers promising applications in improving solubility, metabolic health, and thermogenesis, highlighting the potential of both as therapeutic agents against obesity and related disorders.

## 1. Introduction

Metabolic syndrome is a worldwide health problem driven by multifactorial variables that affect various biological systems in the body. One of the factors that promotes the development of this syndrome is the excessive accumulation of adipose tissue [1]. Adipose tissue hypertrophy is generally caused by an increase in energy intake accompanied by a decrease in energy expenditure, resulting in a positive balance that is evidenced by an excess of lipid storage [2]. Abnormal lipid accumulation affects adipose tissue and hepatic metabolism by increasing the flow of circulating free fatty acids from adipose tissue to the liver and increasing hepatic lipogenesis [2]. Several metabolic pathways are involved in the progression of metabolic abnormalities during the development of obesity, leading to changes in adipose tissue inflammation, insulin resistance and adipogenesis [3]. Intracellular lipid accumulation in organs other than adipose tissue promotes an increase in circulating cytokines, leading to a systemic inflammatory state [2,3]. This systemic inflammation, accompanied by cytokine release in peripheral tissues and intracellular lipid accumulation, decreases insulin sensitivity and promotes vascular proliferation of the media artery by macrophages, which further increases cytokine release [3,4,5,6].

Since the lipotoxicity observed during metabolic syndrome is caused by the abnormal accumulation of lipids in various organs during the development of metabolic syndrome [5], many strategies to combat this disease focus on reducing the amount of circulating and stored lipids, as well as increasing energy expenditure to enhance the utilization of fatty acids as a source of energy. In recent years, there has been increased interest in the beneficial metabolic effects of certain bioactive compounds in foods, such as resveratrol, a polyphenol of the stilbene family found in red grapes [7,8]. Studies have shown that resveratrol improves energy expenditure by activating brown adipose tissue [9,10,11,12] and promoting browning of white adipose tissue [10,12], resulting in increased energy expenditure, decreased lipid accumulation, and improved glucose tolerance [10]. However, there are several bioactive compounds in the diet that may also have metabolic benefits that have not been studied.

Naringenin (NAR) and fisetin (FST), natural flavonoids found in a wide variety of fruits and vegetables, are known for their potent health benefits. Naringenin, found in citrus fruits such as grapefruit and oranges, has potent antioxidant [13] and anti-inflammatory [14,15] properties. It also promotes cardiovascular health by improving HDL cholesterol levels and reducing the concentration of circulating triglycerides [16], as well as improving insulin sensitivity [17]. In addition, naringenin has been shown to reduce cholesterol and lipid biosynthesis [18,19]. Fisetin, found in strawberries, apples and onions, is known for its potent antioxidant [20] and anti-inflammatory properties [21,22]. In addition, there is evidence that fisetin improves insulin sensitivity, decreasing the accumulation of hepatic lipids [23]. Both compounds are being studied for their therapeutic applications and promise important benefits for human health. However, further studies are needed to investigate their ability to regulate lipid accumulation in the liver and adipose tissue to understand their protective properties against obesity.

In the last decade, several effects of dietary bioactive compounds such as flavonoids have been shown to be associated with their ability to modulate the taxonomy of the gut microbiota, which may in part mediate the health benefits of these compounds [24]. It is likely that the biological effects of flavonoids are influenced by the fluctuating availability of these compounds due to their chemical nature, a factor which may limit their biological activity, since they are poorly soluble in water [25].

To overcome these limitations, we propose new co-amorphous phases based on non-covalent interactions with a co-former that stabilizes the active compounds. Amorphous solids lack long-range order and have a notoriously low packaging efficiency and a high potential energy compared to their crystalline counterparts, generally resulting in improved solubility and permeability [26,27], albeit with a reduction in chemical stability. These proposed co-amorphous systems are formed by solid-state molecular interactions (particularly hydrogen bonds) between an active molecule and an innocuous and low-molecular weight compound, such as an amino acid or organic acid, which enhance the stability of the co-amorphous systems by inhibiting the crystallization of individual components [28].

Attempts are being made to modify the solubility of these compounds to determine the effects of these molecules in the body, as well as their interaction with the gut microbiota. However, more research is still needed in this area to understand the potential effects on the host. Therefore, the main aim of the present study was to determine the effect of the CoA bioactive compounds naringenin and fisetin on energy expenditure, lipid metabolism, expression of genes involved in thermogenesis, and gut microbiota in mice fed a high-fat, high-sucrose diet (HFSD).

## 2. Materials and Methods

### 2.1. CoA Flavonoids Synthesis

CoA-flavonoids were obtained at a flavonoid/arginine molar ratio of 1:3 by flash evaporation, using water as solvent for CoA-Fisetin and ethanol/water (9:1) for CoA-Naringenin, while the mixtures were rotated at 80 °C. The resulting solids were characterized by PXRD, FT-IR, NMR and DSC/TGA [29]. The solubility profiles of the CoA-flavonoids and starting materials (NAR and FST) were assayed in buffer solutions of pH 1.2, 4.5 and 6.8 by UV-Vis spectrophotometer S-3100 (Scinco, Seoul, Republic of Korea). The thermodynamic solubilities of the CoA-flavonoids were measured by gradually adding CoA-flavonoids to 0.5 mL of distilled water until the solution reached supersaturation. More information on these components can be found in patent application WO2024116042.

### 2.2. Animal Care and Maintenance

All the protocols and procedures described in this section had been approved on 13 January 2023, by the Animal Care Committee of the National Institute of Medical Sciences and Nutrition, CICUAL-2082-22-23-1, Mexico City. All of the animal procedures described in the experimental section were carried out inside the animal care facility. The first two authors were aware of the assignment of groups in the different phases of the experiment.

To perform this study, we defined the following as inclusion criteria: 8-week-old male C57BL/6 mice weighing 22–25 g; if, at the time of study’s initiation, mice did not meet these parameters, those mice were not included in the study. The animals were confined in cages with 3 or 4 animals per cage. Animals lived with a 12 h light–dark cycle, at 22 °C for 100 days. To promote animal wellbeing, mice were provided with enrichment toys, such as mice tunnels, from the beginning to the end of experiment. The sample size for the animals was calculated using the equation for comparison of two means of available data from previous publications related to flavonoid consumption as a basis; glucose tolerance data were used to calculate the sample size, and the result was 7 animals per group [30,31]. It has been reported that up to 10% of C57BL/6 mice do not respond to HFSD consumption; therefore, to avoid within-group variation, this 10% was taken into account, and the minimum final sample size was 8 mice per group [32], while 9 animals were included in the groups functioning as controls, which were fed AIN93 or HFSD, to increase the statistical power.

During the study, the health of the mice was monitored according to the end point criteria protocol, which had been previously approved by the ethical review committee. Mice were assigned to five different groups of diets: AIN-93 diet (control diet) (*n* = 9), high-fat, high-sucrose diet (HFSD) (*n* = 9), HFSD with 0.2% of CoA-naringenin (*n* = 8), HFSD with 0.2% of -CoA-fisetin (*n* = 8), and HFSD with 0.2% of resveratrol (*n* = 8) as positive control. The HFSD, as compared to the AIN-93 diet, contained twice as much sucrose and lard and was administered in dry form. AIN-93 and HFSD composition is described in Table 1. The CoA percentage was calculated to provide 0.2% of the flavonoid. The control diet provided 65% of its energy from carbohydrates, 15% from lipids and 20% from protein, while the HFSD diet provided 35% of its energy from carbohydrates, 45% from lipids and 20% from protein. Food consumption and the body weight of the mice were measured twice per week. At the end of the study, animals were euthanized after an 8 h fasted period. Whole blood was collected in a centrifuge tube with heparin and the serum was obtained and frozen at −80 °C after centrifugation at 1000× *g* for 10 min. Inguinal subcutaneous WAT, in addition to liver samples, were obtained and immediately frozen in liquid nitrogen. Tissue samples were stored at −80 °C.

### 2.3. Determination of Body Composition

To determine fat and lean-mass content, a quantitative magnetic resonance imaging system (Echo MRI, Houston, TX, USA) was used. Mice were individually placed into a thin-walled plastic tube, with a cylindrical plastic insert added to restrict movement. During the measurement, the animals were briefly subjected to a low intensity (0.05 Tesla) electromagnetic field to measure fat and lean mass. Measurements were performed twice, at baseline and after 90 days after the diet intervention began (*n* = 8–9 mice per group).

### 2.4. Intraperitoneal Glucose Tolerance Test

On day 90, we performed an intraperitoneal glucose tolerance test. Previous to the conducting of the test, the mice were fasted for a short period of time (7 h). All blood samples for serum glucose measurement were obtained from a small tail cut. Measurement of the basal glucose concentration was obtained prior to the intraperitoneal injection; the glucose concentration used to perform this study was of 2 g kg^−1^ body weight. Tail blood samples were obtained at 15, 30, 45, 60 and 120 min after injection. Glucose concentration was measured with the One-Touch Ultra glucometer (Accu-Check Sensor, Roche Diagnostics). (For this procedure, *n* = 8–9 mice per group.)

### 2.5. Indirect Calorimetry

On day 85, an indirect calorimetry test was carried out using a Comprehensive Lab Animal Monitoring System (Columbus Instruments, Columbus, OH, USA). Mice were individually housed, and measurements were conducted during a 24 h period. The test was carried out at 22 °C under a 12 h/12 h light–dark cycle. During the performance of the test, food was only available from 19:00 to 7:00 h, and the animals always had water ad libitum. The mice were weighed before the test was performed (*n* = 8–9 mice per group).

### 2.6. Serum Measurements

Serum glucose, cholesterol, LDL cholesterol, creatinine, urea, aspartate aminotransaminase (AST) and alanine transaminase (ALT) were measured with a COBAS c111 analyzer (Roche, Basel, Switzerland). Serum adiponectin and insulin concentrations were measured using Merck Millipore ELISA kits (Merck Millipore, Darmstadt, Germany) and leptin levels were measured using a commercial enzyme-linked immunosorbent assay (Thermo fisher Scientific, Waltham, MA, USA) (*n* = 8–9 mice per group).

### 2.7. Western Blot Analysis

Subcutaneous and epididymal WAT extracts were prepared by homogenizing the tissue in an RIPA buffer containing PBS buffer, SDS, sodium deoxycholate, sodium azide, NP-40, and protease and phosphatase inhibitors. Total protein was obtained by centrifugation for 20 min. The proteins were separated by SDS-polyacrylamide gel electrophoresis and transferred to polyvinylidene difluoride membranes (Bio Rad Laboratories, Hercules, CA, USA). The membranes were incubated with the specific antibodies anti-Uncoupled protein 1 (UCP1), anti-Peroxisome proliferator-activated receptor gamma coactivator 1-alpha (PGC1α), and anti-GAPDH, which were obtained from Abcam (Cambridge, UK). Anti-GAPDH was used as a loading control to normalize protein abundance. The protease and phosphatase inhibition cocktail was purchased from Roche, (Mannheim, Germany). To determine protein content, the Immobilon Western chemiluminescent HRP substrate detection system was used; it was obtained from Millipore (Temecula, CA, USA). The chemidoc photo-documenter system (Bio-Rad Laboratories, Inc., Hercules, CA, USA) was used to record the blots’ images. Digital images were analyzed using ImageJ 1.51 (100) 2015 digital imaging processing software (*n* = 4–6 animals per group).

### 2.8. Histological Analysis

Subcutaneous, epididymal and brown adipose tissue samples were collected from the mice to perform histological analysis with the hematoxylin and eosin (H/E) staining. The samples were preserved in formalin to stabilize the tissue and prevent it from decaying. Afterwards, the samples experienced a dehydration process to remove their water content, and were then fixed in paraffin for sectioning. The tissues were cut into slices and then stained with specific dyes. The samples were then mounted on glass slides under coverslips for image capture using a microscope. Subsequently, these images were analyzed using ImageJ software to quantify different parameters, for further analysis.

### 2.9. Lipid Content Determination in Liver

The Folch method was used to extract the lipids from biological samples. The sample was first mixed with a solvent mixture of chloroform and methanol to disrupt cell membranes and release lipids into the solvent. The sample was then shaken extensively to ensure extraction. Subsequently, methanol was added to induce separation between the chloroform and the aqueous phase to dissolve the lipids in chloroform. Finally, the samples were dried with nitrogen to concentrate the lipid extracts. The concentration of fatty acids in the liver was determined by gas chromatography, and the samples were injected into an Agilent DB-225MS (Santa Clara, CA, USA) 30 m × 0.25 mm × 0.25 µm analytical column coupled to a flame detector (FID) for analysis. An Agilent 6850 Series II gas chromatograph (Santa Clara, CA, USA) coupled to an auto-sampler (Agilent 6850, Santa Clara, CA, USA) was used. Total hepatic triglycerides and cholesterol concentrations were measured with triglycerides and Total Cholesterol FS Kits (DiaSys, Diagnostic Systems, Hoizheim, Germany). Lipid concentrations were calculated from the samples’ absorbance, measured with a spectrophotometer (Bio Rad Laboratories, Hercules, CA, USA) (*n* = 8–9 mice per group).

### 2.10. Microbiota Analysis

At the end of the study, a fecal sample was collected from all animals treated with the different diets. Fecal samples were stored in an RNA later QIAGEN (Hilden, Germany), frozen at −80 °C. DNA extraction was carried out using a QIAamp Power Fecal Kit (QIAGEN, Hilden, Germany) according to the manufacturer’s instructions.

The Illumina kit was used for DNA sequencing. Variable regions 3–4 of the 16S rRNA gene were amplified using specific forward (5′ TCGTCGGCAGCGTCAGATGTGTATAAGAGACAGCCTACGGGNGGCWGCAG3) and reverse primers (5′ GTCTCGTGG GCTCGGAGATGTGTATAAGAGACAGGACTACHVGGTATCTAATCC 3) containing the Illumina adapter overhang nucleotide sequences. Ampure XP bits were used to purify the 16S V3-V4 amplicons, and quantified on Qiaxcel (QIAGEN, Hilden, Germany). The amplicon size was approximately 550 bp. An index PCR was then carried out to attach dual indices using a Nextera XT v2 Kit (Illumina, San Diego, CA, USA) (The amplicon size was approximately 610 bp, and the concentration of double-stranded DNA was measured using a fluorometer Qubit 3.0 with a high-sensitivity kit (Thermo Fisher Scientific, Waltham, MA, USA). The final amplicon library was pooled in equimolar concentrations. Sequencing was performed on the IlluminaMiSeq platform (MiSeq Reagent Kit V.3 (Illumina, San Diego, CA, USA), 600 cycles) at 10 pm with 20% Phyx infection, according to the manufacturer’s instructions, to generate paired end reads of 300 bases in length in each direction.

For taxonomic composition analysis, Custom C# and python scripts in the Quantitative Insights IntoMicrobial Ecology (QIIME) software pipeline v 2-2022.2 were used to process the sequencing files. The sequence outputs were filtered for low-quality sequences (defined as any sequences that were <200 bp or >620 bp, sequences with any nucleotide mismatches to either the barcode or the primer, sequences with an average quality score of <30, and sequences with ambiguous bases > 0). Sequences were checked for chimeras, and chimeric sequences were filtered out. The analysis started by clustering sequences within a certain percentage of sequence-similarity into Amplicon Sequences Variances (ASVs). ASVs were picked using the SILVA database. Thus, the reads were assigned to their different taxonomic levels. Alpha diversity measurements (Shannon, South Melbourne, Australia) were calculated. Bray–Curtis PCoA analysis was performed to determine similarity; ANOSIM, a permutational multivariate analysis of variance, was used to determine the statistically significant clustering of groups based upon microbiota structure distances (*n* = 7 mice per group).

### 2.11. Statistical Analysis

Results are presented as the means ± SEMs. Analysis of more than two groups was performed by one-way ANOVA followed by Fisher’s protected least-square difference test. Differences were considered significant at *p* < 0.05 (GraphPad Prism San Diego, La Jolla, CA, USA).

## 3. Results

### 3.1. Enhanced Solubility and Distinct Properties of Co-Amorphous Naringenin–Fisetin Systems

**Figure 1:**
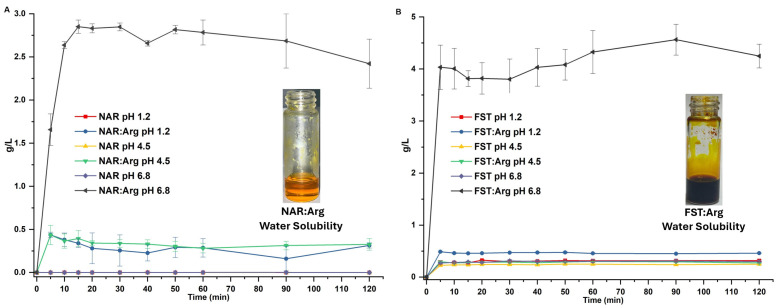
Solubility of CoA flavonoids. Solubility graphs of (**A**) naringenin (NAR) and CoA-naringenin (NAR:Arg) and (**B**) fisetin (FST) and CoA-fisetin (FST:Arg) at different pH values. The image in each graph shows the solubility of CoA-naringenin and CoA-fisetin in water before plasticization. Results are shown as mean ± S.E.M.

In the analysis of co-amorphous systems of naringenin and fisetin, various techniques revealed distinct characteristics when compared to their crystalline starting materials—fisetin, naringenin and arginine. Powder X-ray diffraction (PXRD) showed that the co-amorphous systems displayed broad, featureless peaks characteristic of amorphous halos, in contrast to the sharp, well-defined peaks of the crystalline materials. Fourier-transform infrared spectroscopy (FT-IR) revealed significant shifts in the O-H stretching vibrations of the flavonoids within the co-amorphous systems, suggesting that hydroxyl groups, rather than carbonyl groups, are responsible for forming intermolecular interactions with arginine. Differential scanning calorimetry and thermogravimetric analysis (DSC-TGA) indicated that the narigenin (NAR) system underwent an endothermic event at 91.42 °C and a glass transition at 88.3 °C, followed by decomposition. Meanwhile, fisetin (FST) exhibited dehydration at 95.5 °C, a glass transition at 111.32 °C, and decomposition at 238 °C. In solubility tests, both co-amorphous systems dissolved rapidly and completely at pH 6.8, showing significantly improved solubility compared to their individual components, with slight improvements observed at pH 1.2 and 4.5 (Figure 1A,B). The increased viscosity from the co-amorphous material did not result in supersaturation. Overall, these findings underscore the enhanced solubility of the co-amorphous systems relative to their crystalline counterparts, as detailed in patent document WO20241160427. (The abbreviation NAR:Arg corresponds to CoA-naringenin, and FST:Arg corresponds to CoA-fisetin.)

### 3.2. Consumption of CoA-Naringenin Decreases Fat-Mass Gain in Mice Consuming an HFSD

**Figure 2:**
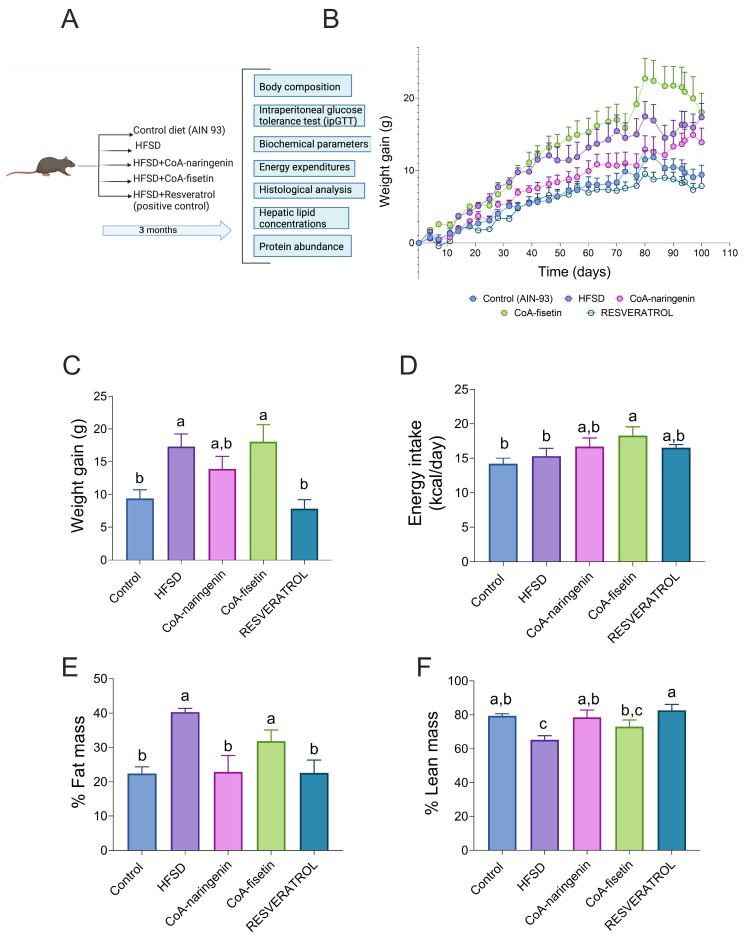
Effects of CoA-flavonoids in body weight, food intake, percentage of fat mass and percentage of lean mass, in mice with AIN-93 diet and HFSD. (**A**) Experimental design. (**B**) Body weight gain during the study, (**C**) body weight at the end of the study, (**D**) energy intake at the end of the study and (**E**) percentages of fat mass and (**F**) lean mass at the end of the study. Results are shown as means ± S.E.M. (*n* = 8–9 mice per group). One-way ANOVA was performed; letters indicate differences between groups (*p* = 0.05) a > b > c.

To assess the effect of CoA flavonoids on the physiological parameters related to metabolic syndrome and obesity, weight gain and body composition were measured over the three-month study period (Figure 2A). The results showed, as expected, that the HFSD group gained significantly more weight (84%) compared to the control group. Mice that consumed CoA-fisetin exhibited a similar increase in body weight relative to the HFSD group. However, mice that consumed CoA-naringenin along with HFSD gained 46% less weight than the HFSD group (Figure 2B), though by the end of the study, the difference between the CoA-naringenin group and the HFSD group was not statistically significant (Figure 2C). As anticipated, mice fed resveratrol with HFSD gained less weight compared to both the HFSD group (121%) and the control group (20%) (Figure 2C). Food consumption varied only in the CoA-fisetin group, indicating that changes in body weight in the other groups were not related to food intake (Figure 2D). These results suggest that CoA-naringenin consumption tends to reduce weight gain when combined with an HFSD. Based on this finding, a body composition analysis was conducted. In the fat-mass analysis, the HFSD + CoA-naringenin group gained 45% less fat mass compared to the HFSD group, which was similar to the fat-mass gain of the resveratrol group. Mice in the CoA-fisetin group gained the same percentage of fat mass as the HFSD group (Figure 2E). No significant difference was observed in fat-mass gain between the CoA-naringenin and resveratrol groups. Lean-mass patterns were similar across all groups (Figure 2F). These findings suggest that CoA-naringenin primarily reduces fat-mass gain.

### 3.3. CoA-Naringenin Improves Cholesterol Metabolism in Obese Mice

**Figure 3:**
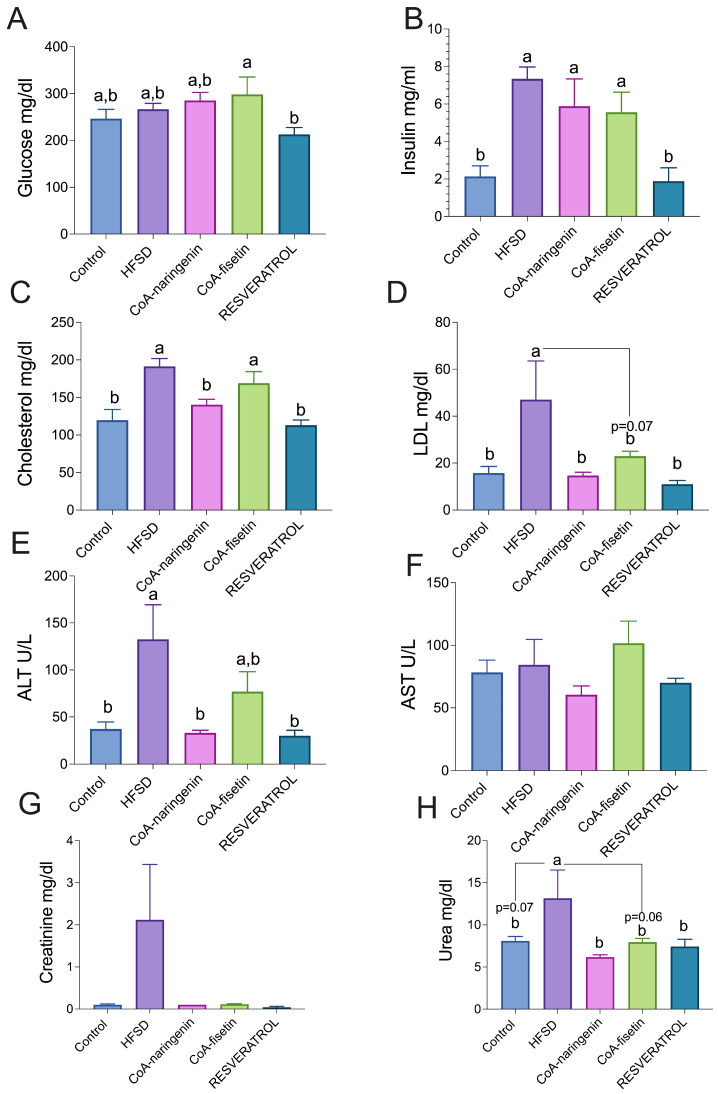
Effects of CoA-flavonoids in serum concentrations of insulin, glucose, cholesterol, hepatic transaminases, urea and creatinine in mice with AIN-93 diet and HFSD. (**A**) Glucose, (**B**) Insulin, (**C**) Total Cholesterol, (**D**) LDL cholesterol, (**E**,**F**) Hepatic Transaminases, (**G**) Creatinine and (**H**) Urea. All parameters were measured at the end of the study by COBAS c111 Analyze; Insulin was measured using an EKISA Kit. Results are shown as the means ± S.E.M. (*n* = 8–9 mice per group). One-way ANOVA was performed; letters indicate differences between groups (*p* = 0.05) a > b. Results are shown as means ± S.E.M.

To describe the metabolic profile of the mice that consumed different CoA flavonoids, various biochemical parameters were measured. Glucose (Figure 3A) and serum insulin (Figure 3B) levels did not show significant differences between the mice fed only a high-fat, high-sugar diet (HFSD) and those with diets supplemented with CoA flavonoids (naringenin and fisetin). However, significant differences were observed with resveratrol (Figure 3A,B). Regarding hepatic metabolism, CoA-naringenin consumption notably reduced total and LDL cholesterol levels compared to the HFSD group, indicating an improvement in cholesterol metabolism when CoA-naringenin was included in the diet (Figure 3C). Mice fed CoA-fisetin showed a reduction in LDL cholesterol compared to the HFSD group but this did not reach statistical significance (*p* = 0.07). As expected, resveratrol-fed mice exhibited a reduction in both total and LDL cholesterol, which is similar to the effect of CoA-naringenin (Figure 3D). There were no significant differences between these two groups and the control group. The hepatic inflammation determination only showed that CoA-naringenin decreased significant ALT; however, AST did not show a significant difference compared to the control group (Figure 3E,F). Similarly, creatinine levels did not vary significantly between groups (Figure 3G). However, urea levels, which were elevated by HFSD consumption, were reduced with CoA-naringenin and resveratrol consumption (Figure 3H). The consumption of CoA-fisetin showed a tendency to lower LDL urea, but this was not statistically significant (*p* = 0.06).

### 3.4. CoA-Flavonoids Increase VO_2_: Consumption Improves Metabolic Flexibility and Glucose Tolerance in Mice Fed with HFSD

**Figure 4:**
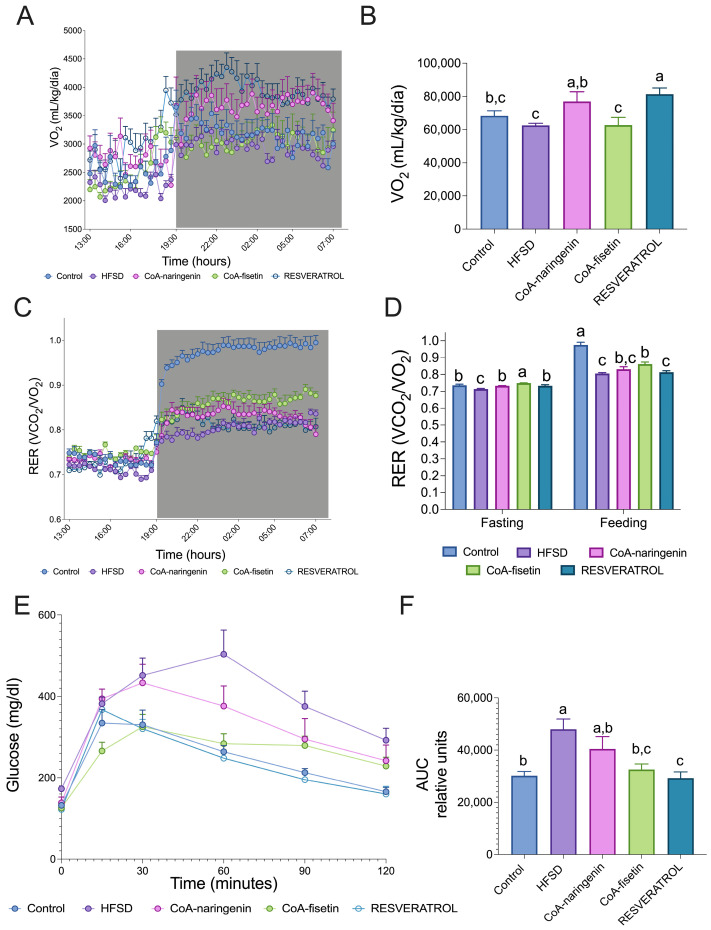
Measurement of energy expenditure and glucose tolerance in mice with an AIN-93 diet and HFSD with or without CoA-flavonoids. determined by indirect calorimetry. (**A**) Oxygen consumption (VO_2_ mL/h) and (**B**) total oxygen consumption (VO_2_ mL/h), (**C**) RER time course (VCO2/VO_2_) and (**D**) RER in fasting and feeding period, (**E**) Glucose tolerance test (GTT) and (**F**) area under the curve of GTT fed with a control or HFSD with or without CoA-flavonoids. The slope and intercept in mL/h or Kcal/h are indicated for each condition (*n* = 8–9 per group). One-way ANOVA was performed; letters indicate differences between groups (*p* = 0.05) a > b > c. Results are shown as means ± S.E.M.

One essential parameter related to energy expenditure is the volume of oxygen consumption (VO_2_). To assess the effect of CoA flavonoids on energy consumption, indirect calorimetry was measured (Figure 4A). VO_2_ increased in mice fed with CoA-naringenin compared to those fed with a high-fat, high-sucrose diet (HFSD) (Figure 4B), indicating a rise in energy expenditure. This could explain the reduced fat-mass accumulation observed with CoA-naringenin consumption, even in the presence of an HFSD. In contrast, while resveratrol also increased VO_2_, CoA-fisetin did not significantly alter VO_2_ compared to the HFSD-only group (Figure 4B).

Another parameter measured during indirect calorimetry was the respiratory exchange rate (RER). HFSD consumption resulted in metabolic inflexibility, with energy being derived almost exclusively from lipid oxidation (RER: 0.7–0.8) (Figure 4C), indicating an inability to oxidize carbohydrates, even when they are available in the postprandial period. By contrast, normal diet consumption typically results in an RER close to 1.0 during the postprandial phase. In this experiment, CoA-naringenin and fisetin improved RER during the fasting period, enhancing the metabolic flexibility impaired by HFSD consumption. In the postprandial phase, mice fed CoA-fisetin showed a significant increase in RER, while CoA-naringenin only showed a rising trend that was not statistically significant (Figure 4D). Notably, resveratrol did not induce this increase, which was only observed with CoA-flavonoid consumption (Figure 4D).

During obesity, glucose tolerance is impaired, leading to reduced insulin sensitivity and a higher risk of diabetes. To evaluate glucose metabolism, we performed an intraperitoneal glucose tolerance test (iGTT) on all experimental groups. Serum glucose levels were measured at 15, 30, 60, 90 and 120 min post-glucose injection (Figure 4E). As expected, HFSD increased glucose concentrations compared to the control group. However, mice fed CoA-fisetin showed a tendency to improve glucose tolerance, compared with those consuming only HFSD (Figure 3F and Figure 4E). Fisetin was associated with an improvement in glucose tolerance, compared with those consuming only HFSD (Figure 3F and Figure 4E). The area under the curve (AUC) analysis further confirmed that CoA-fisetin-consumption-associated glucose levels tend to be similar to those of mice on a control diet, despite the mice consumed HFSD (Figure 4F).

### 3.5. CoA-Flavonoids Decrease Hepatic Lipids in Diet-Induced Obesity Model

**Figure 5:**
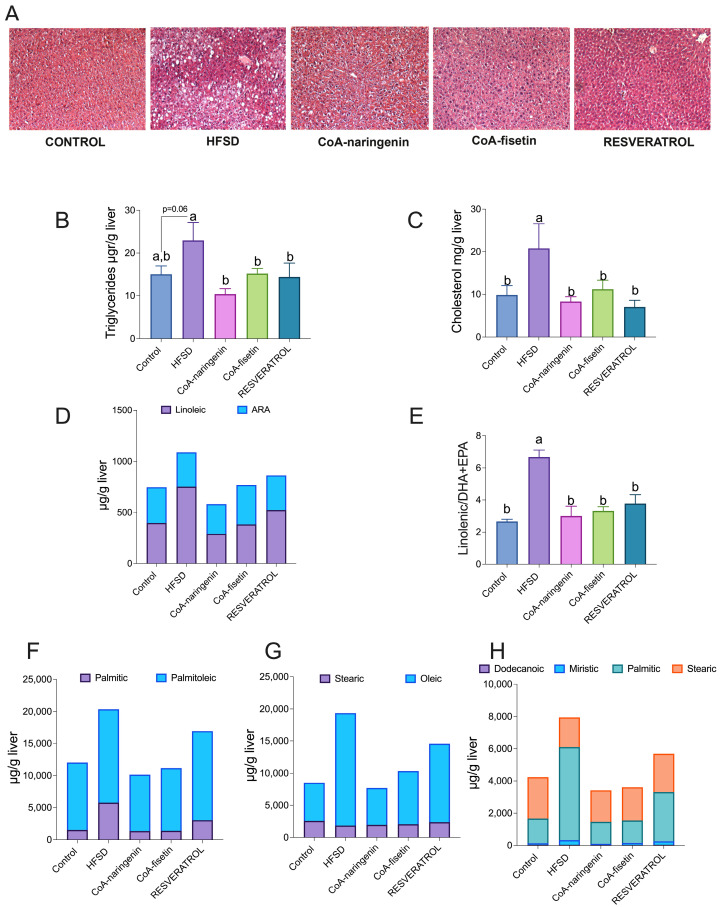
Effect of CoA-flavonoids on hepatic lipids. (**A**) H/E staining of liver; (**B**) quantification of total hepatic triglycerides and (**C**) total cholesterol; (**D**) hepatic determination of linolenic acid, DHA, and EPA and (**E**) linolenic acid, as well as the DHA+EPA ratio; (**F**) quantifications of palmitic and palmitoleic acid, (**G**) stearic and oleic acid and (**H**) saturated fatty acids, as determined by gas chromatography. All of the quantifications were determined for all mice fed with a control diet or HFSD, with or without CoA-flavonoids. Lipids samples were obtained through the Folch method. Results are shown as the means ± S.E.M. (*n* = 8–9 mice per group). One-way ANOVA was performed; letters indicate differences between groups (*p* = 0.05) a > b. Results are shown as means ± S.E.M.

The pathophysiological effects of obesity on the liver include lipid accumulation in peripheral tissues, leading to abnormal fat deposition, insulin resistance, hepatic inflammation and steatosis. Given that the liver is a key metabolic organ, and bioactive compounds are known to improve lipid metabolism, we investigated hepatic morphology and lipid profiles in mice fed with CoA-flavonoids. To assess hepatic morphology, H/E staining was performed. As shown in Figure 5A, livers of mice fed a high-fat, sugar-rich diet (HFSD) displayed increased fat accumulation, evidenced by prominent lipid droplets, while the control group showed no such droplets, indicating normal lipid distribution. In contrast, mice treated with CoA-naringenin and CoA-fisetin exhibited fewer lipid droplets compared to the HFSD group, with results instead resembling the lipid profile of the control diet group.

To confirm these findings, we extracted liver lipids using the Folch method and quantified triglycerides and total cholesterol. Mice on the HFSD alone had elevated liver triglyceride levels, while the CoA-naringenin and CoA-fisetin groups showed significant reductions of 54% and 32%, respectively, compared to the HFSD group (Figure 5B), suggesting improved lipid metabolism. Additionally, liver cholesterol levels in the CoA-naringenin and CoA-fisetin groups were reduced by 61% and 46%, respectively, compared to the HFSD group (Figure 5C). Resveratrol also lowered liver lipid content (Figure 5C).

Given the reduction in triglyceride levels observed with CoA-flavonoid consumption, we further analyzed liver lipid composition by measuring different fatty acid concentrations. The HFSD group exhibited higher levels of linolenic acid in both absolute and relative terms compared to all other groups (Figure 5D,E). In particular, the CoA flavonoid and resveratrol groups showed higher concentrations of DHA and EPA relative to linolenic acid, more closely resembling the normal diet group. This is significant, as DHA and EPA are known for their anti-inflammatory properties [33,34], which is consistent with previous reports on fisetin and naringenin (Figure 5E).

Regarding other fatty acids, the HFSD group had elevated levels compared to all other groups (Figure 5F,G). Mice fed only the HFSD had higher palmitic acid concentrations, whereas those treated with CoA-flavonoids showed lower palmitic acid levels and higher palmitoleic acid levels relative to palmitate. Saturated fatty acids were reduced with the consumption of CoA-naringenin and CoA-fisetin, resulting in a lipid profile similar to that of the control group (Figure 5H). These results suggest that CoA flavonoid consumption improves lipid metabolism by reducing hepatic lipid content and increasing the concentration of anti-inflammatory fatty acids.

### 3.6. CoA-Flavonoids Diminish Adipocyte Size, Increasing Thermogenic Markers

Body composition analysis indicates that mice fed with CoA-naringenin gained a lower percentage of fat mass despite consuming a high-fat, high-sucrose diet (HFSD), while maintaining a quantity of lean mass comparable to mice on a control diet. Previous studies have shown that some bioactive compounds can activate thermogenesis [35,36], particularly in inguinal white adipose tissue, inducing a shift towards “beiging” tissue, which increases energy expenditure [35]. This beiging process improves glucose metabolism and fatty acid oxidation.

**Figure 6:**
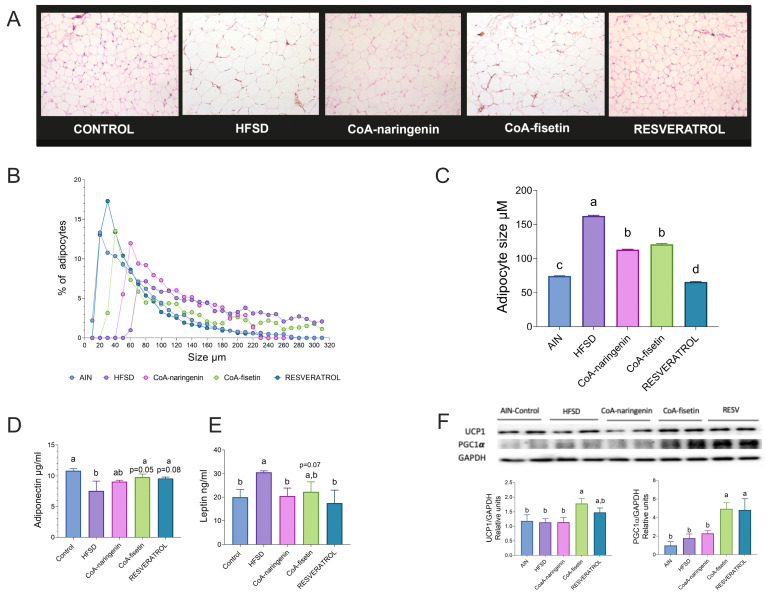
Effect of CoA-flavonoids in white and brown adipose tissue metabolism. (**A**) H/E staining of WAT. (**B**) Size quantification of adipocytes and the (**C**) mean size of WAT, quantification performed by adiposoft. (**D**) Serum concentrations of adiponectin and (**E**) leptin, measured by an ELISA Kit. (**F**) Immunoblotting and densitometric analysis of UCP-1 and PGC1-α from iWAT, fed with a control and HFSD with or without CoA-flavonoids. Results are shown as the means ± S.E.M. (*n* = 4 per group). One-way ANOVA was performed; letters indicate differences between groups (*p* = 0.05) a > b > c. Results are shown as means ± S.E.M.

To investigate this, we analyzed the morphology and size of adipocytes using H/E staining, as shown in Figure 6A. As expected, adipocytes from mice fed an HFSD were larger compared to those on the control diet. However, in the CoA-naringenin and resveratrol groups, adipocytes were significantly smaller. Notably, CoA-naringenin-treated mice had a higher percentage of small adipocytes compared to the HFSD group, a trend also seen in the CoA-fisetin group (Figure 6B,C). Using images from H/E staining, the mean adipocyte size was calculated for all groups. Mice fed CoA-naringenin had a mean adipocyte size of 113 µM, 30% smaller than those in the HFSD group (162 µM) (Figure 6C). Similarly, CoA-fisetin reduced adipocyte size to 120 µM, even with HFSD consumption. Resveratrol led to the smallest adipocytes, with an average size of 65.7 µM, significantly smaller than all other groups (Figure 6C).

Adipose tissue plays a key role in metabolic regulation by releasing adipokines such as adiponectin, which controls glucose and lipid oxidation, and leptin, which regulates appetite. In obesity, dysregulation of these adipokines is common, leading to leptin resistance associated with elevated leptin levels and reduced adiponectin, impairing metabolism and appetite regulation [37,38]. To assess white adipose tissue function, we measured adiponectin and leptin concentrations. In mice on an HFSD, adiponectin levels were significantly lower compared to the control group. Although CoA-naringenin reduced adiponectin levels, the difference was not statistically significant. However, CoA-fisetin and resveratrol preserved adiponectin levels similar to the control group (Figure 6D). This suggests that CoA-flavonoid supplementation mitigates the impact of HFSD on adiponectin levels. Leptin levels in the CoA-naringenin and resveratrol groups were lower compared to the HFSD group and nearly identical to the control group (Figure 6E). CoA-fisetin also showed a reduction in leptin levels, though this result was not statistically significant (Figure 6E).

Based on the observed improvements in metabolic health, we further evaluated the thermogenic activation potential of these compounds by assessing two beiging markers, uncoupled protein 1 (UCP1) and peroxisome proliferator-activated receptor gamma coactivator 1-alpha (PGC-1α), through Western Blot analysis in WAT. Consumption of CoA-fisetin and resveratrol increased PGC-1α protein abundance compared to the HFSD group (Figure 6F); however, only CoA-fisetin raised the UCP1 concentration, since, although resveratrol tended to increase UCP1, this was not statistically significant. We did not observe an effect of CoA-naringenin on beiging markers. These results indicate that consumption of CoA-fisetin promotes mitochondrial biogenesis, increasing PGC-1α, a key factor in activating thermogenesis and promoting beiging differentiation.

### 3.7. Changes in the Intestinal Microbiota Are Partially Modulated by the Consumption of CoA-Flavonoids

**Figure 7:**
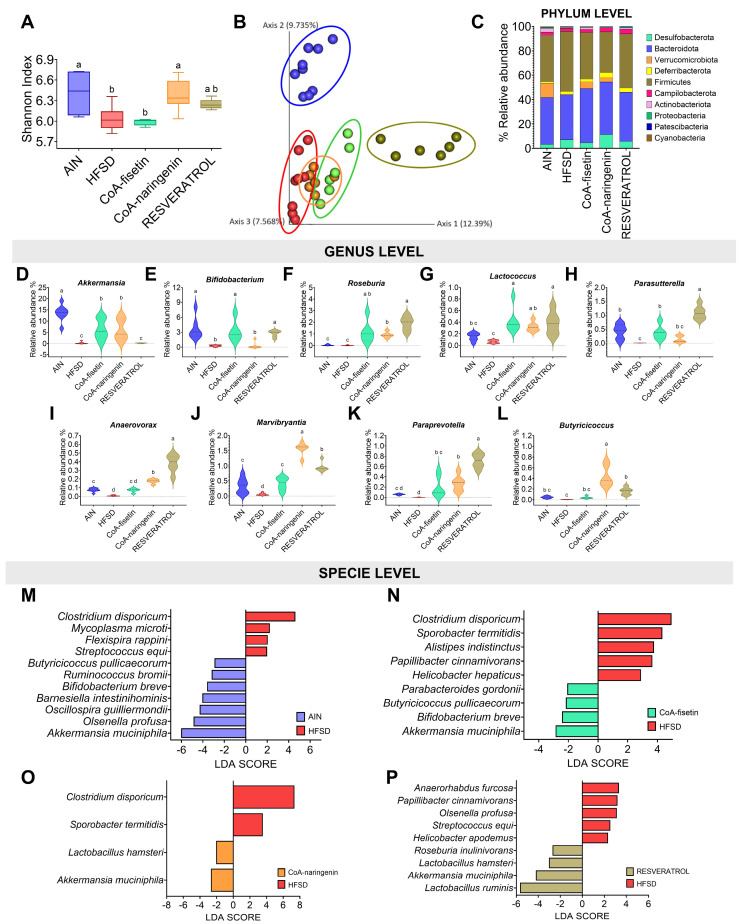
Effect of CoA-flavonoids on intestinal microbiota. (**A**) Alpha diversity by Shannon index. (**B**) Beta diversity by Jaccard. (**C**) Relative abundance at the phylum level and relative abundance at the genus level for (**D**) *Akkermansia*, (**E**) *Bifidobacterium*, (**F**) *Roseburia*, (**G**) *Lactococcus*, (**H**) *Parasutterella*, (**I**) *Anerovorax*, (**J**) *Marvibryantia*, (**K**) *Paraprevotella* and (**L**) *Butyricicoccus*. Linear discriminant analyses comparing (**M**) C and HFSD groups, (**N**) Fisetin and HFSD groups, (**O**) Naringenin and HFSD groups and (**P**) Resveratrol and HFSD groups. Letters indicate differences between groups (*p* = 0.05) a > b > c > d. Results are shown as means ± S.E.M.

When evaluating the gut microbiota, a decrease in α-diversity was observed with the consumption of an HFSD, compared with the control group (C). However, the addition of compounds such as CoA-naringenin or resveratrol partially restored this diversity (Figure 7A). In terms of β-diversity, there was a marked dissimilarity between group C and those on the HFSD diet, although a slight change was observed with CoA-fisetin supplementation, and an even more pronounced effect with resveratrol (PERMANOVA, *p* < 0.001) (Figure 7B). At the phylum level, the intake of CoA-naringenin or CoA-fisetin increased the abundance of Verrucomicrobiota to levels similar to those of the control group, which is associated with improved glucose tolerance. Resveratrol, on the other hand, restored the balance between Bacteroidetes and Firmicutes, resembling the profile of the control group (Figure 7C). At the genus level, significant increases were observed in Akkermansia, Bifidobacterium, Roseburia, Lactococcus, Parasutterella, Anaerovorax, Marvinbryantia, Paraprevotella and Butyricicoccus across different bioactive compounds. These genera are related to beneficial effects on metabolism, maintenance of the integrity of the intestinal epithelium and anti-inflammatory properties (Figure 7D–L). At the species level, an increase in bacteria associated with metabolic endotoxemia and liver inflammation, such as *Streptococcus equi* and *Mycoplasma microti*, was observed in the HFSD group (Figure 7M). In contrast, CoA-fisetin, CoA-naringenin, or resveratrol increased the abundance of *Akkermansia muciniphila*. CoA-fisetin also promoted the growth of butyrate-producing bacteria such as *Butyricicoccus pullicaecorum* and *Bifidobacterium breve*. In the case of resveratrol, an increase in *Roseburia inulinivorans* was observed, which is associated with improved glycemic control (Figure 7O,P).

### 3.8. CoA-Flavonoids Improve Glucose and Lipid Metabolism, Modulating Intestinal Microbiota

**Figure 8:**
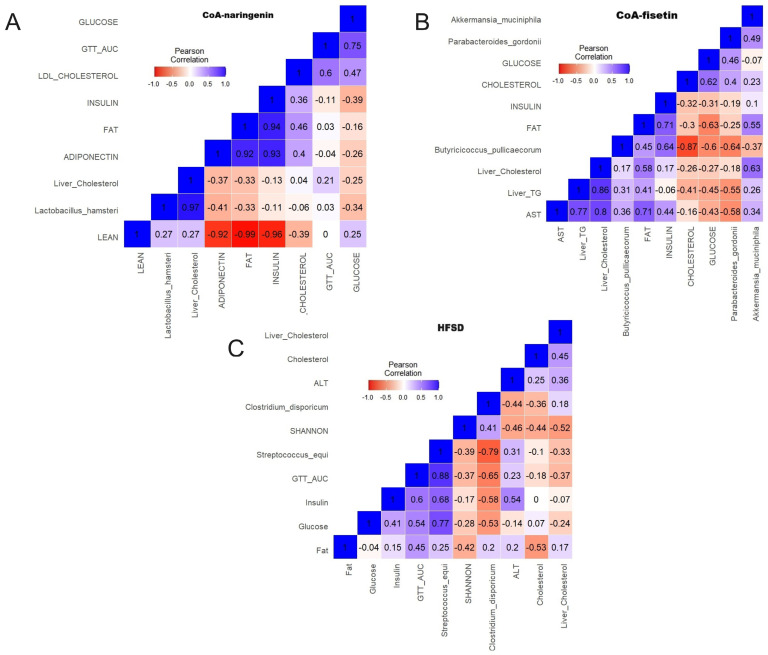
Correlogram of the intestinal microbiota and metabolic markers with CoA-flavonoids consumption. (**A**) Correlogram of CoA-naringenin consumption. (**B**) Correlograms of CoA-fisetin consumption and of (**C**) HFSD consumption. (Pearson correlation.).

The correlogram analysis showed that the consumption of CoA-naringenin and CoA-fisetin increased the abundance of *Akkermansia muciniphila*, which exclusively had a significant association with the decrease of the area under the curve of the ipGTT (Figure 8A,B). Additionally, consumption of CoA-fisetin increased the abundance of *Butyricicoccus pullicaecorum* and *Bifidobacterium breve*,which showed a negative correlation with serum and liver total cholesterol concentration (Figure 8B). These correlations were not observed with the consumption of HFSD; however, it was shown that the consumption of this diet increased the abundance of *Streptococcus equi*, which correlated positively with the serum concentrations of glucose and insulin, as well as with an increase in the area under the curve of the ipGTT (Figure 8C).

## 4. Discussion

One critical element of this study involved increasing the solubility of flavonoids. The development of co-amorphous systems significantly improves solubility, which could improve the absorption of compounds, a factor which is often a limitation in bioactive compounds [39,40]. Despite the transition from the crystalline to the amorphous state, increasing the solubility, there is no evidence that this modification has biological effects, or as to whether these effects have an impact on metabolic diseases or on the gut microbiota [39]. Improved solubility may have significance for pharmaceutical applications [41], where flavonoids such as naringenin and fisetin may offer new therapeutic approaches to address metabolic syndrome.

The present study shows the potential of co-amorphous bioactive compounds, in particular flavonoids such as CoA-naringenin and CoA-fisetin, to reverse or attenuate some metabolic alterations associated with the development of obesity. Because of its widely known beneficial effects on health, unmodified resveratrol was used as a comparison to determine the biological effects of CoA-flavonoids. One of the main findings of this study was the reduction of fat-mass gain and improvement of muscle mass maintenance in the mouse model of obesity, particularly through CoA-naringenin. Compared to resveratrol, CoA-naringenin decreased fat by the same magnitude, but resveratrol consumption decreased weight gain more efficiently than did CoA-naringenin. Furthermore, in terms of hepatic metabolism, CoA-naringenin reduced serum cholesterol and hepatic transaminases, markers that usually indicate metabolic stress in obesity. Naringenin has been recognized as a potent antioxidant with anti-inflammatory properties [13,42], one which has been shown to reduce fatty liver inflammation by regulating the NLRP3/NF-κB pathway [43]. Interestingly, this CoA-flavonoid was associated in this study with an improvement of lipid metabolism in the liver, reducing its accumulation, but also modifying its lipid composition, in particular increasing EPA and DHA, which have anti-inflammatory effects [33,34,44], suggesting a possible role of this CoA-flavonoid in the treatment of conditions such as Metabolic Dysfunction-Associated Steatotic Liver Disease (MASLD). The effects of CoA-naringenin on hepatic lipid metabolism were similar to those observed with resveratrol, in both decreasing total and LDL cholesterol and transaminase concentrations and also increasing hepatic EPA and DHA concentrations. These biological effects are consistent with previous findings on resveratrol consumption in combination with a high-fat, high-sugar diet (HFSD) [45], including the determination of its anti-inflammatory effects on the liver [43,46].

Although fisetin is best known for its anti-inflammatory effects on the liver and immune system [21,22], there are currently no studies examining its impact on energy expenditure, glucose metabolism, or benefits to adipose tissue when consumed in conjunction with an HFSD. Interestingly, despite the finding that CoA-fisetin consumption increased food intake, weight gain, fat mass and some serum biochemical parameters, this CoA-flavonoid produced some beneficial effects. Among these changes, there was a significant increase in the respiratory exchange ratio (RER), suggesting that the use of carbohydrates as an energy source improved the metabolic inflexibility induced by the consumption of an HFSD, and there was a decrease in glucose intolerance. In addition, consumption of CoA-fisetin did not significantly increase energy expenditure; however, there was a reduction in adipocyte size. This was accompanied by an increase in UPC1 and PGC1-α protein content that could be associated with an improvement in adipose tissue functionality, as supported by an increase in adiponectin concentrations and a tendency to decrease serum leptin. The increase of these proteins could explain the diminished weight gain in this experimental group at the end of the study. However, despite the fact that CoA-fisetin showed some metabolic effects, which were mainly observed in liver and adipose tissue, resveratrol showed a significantly greater capacity to reduce the effects of the consumption of an HFSD.

Noteworthily, in terms of energy expenditure, resveratrol was associated with higher oxygen consumption than even the control group, but did not differ in energy expenditure from that produced by the consumption of CoA-naringenin, despite the intake of an HFSD diet. A key factor related to obesity is the expansion of adipose tissue, which is often associated with an increase in adipocyte size. In this study, white adipose tissue (WAT) showed a reduction in size in comparison to the WAT of HFSD-fed mice, following the consumption of CoA-naringenin. Previous studies have reported that naringenin consumption activates the thermogenic program in WAT, stimulating lipolysis in human adipocytes [47,48,49]; however, despite the fact that CoA-naringenin did not increase thermogenic markers, there was a reduction in total body fat content and a decrease in serum leptin, indicative of functional adipocytes.

One of the most interesting aspects of this study is the potential role of the gut microbiota in mediating the observed metabolic changes of bioactive compounds. While the α-diversity of the gut microbiota decreased with HFSD consumption, compounds such as CoA-naringenin, CoA-fisetin and resveratrol partially restored it. However, these bioactives were unable to completely restore the β-diversity altered by HFSD. Interestingly, resveratrol supplementation resulted in a different gut microbial profile compared to CoA-flavonoids, as it mainly restored the balance between Bacteroidetes and Firmicutes, a hallmark of a healthier gut microbiota, and in this, resembled the control group. This differential effect highlights the unique mechanisms through which resveratrol modulates the microbiota, warranting further exploration of its role compared to other CoA-flavonoids. Increased beneficial populations, including Akkermansia and Bifidobacterium, contribute to improved intestinal health and metabolic function [50]. In particular, these interventions favored the growth of beneficial microbial populations such as *Akkermansia muciniphila*, a bacterium associated with improved glucose tolerance and insulin sensitivity, due to its capacity to maintain intestinal barrier integrity and reduce systemic inflammation [51,52,53]. In addition, CoA-fisetin and CoA-naringenin increased the abundance of *Butyricicoccus pullicaecorum*, a butyrate-producing bacterium which has been linked to anti-inflammatory properties and improved glycemic control [54].

Furthermore, resveratrol uniquely increased the abundance of *Roseburia inulinivorans*, another butyrate producer [55], further improving glycemic control. These changes in microbiota composition correlated with some metabolic markers: *Akkermansia muciniphila* showed a significant negative correlation with glucose intolerance, while HFSD-associated *Streptococcus equi*, related to inflammation and metabolic endotoxemia, showed a positive correlation with hyperglycemia and insulin resistance. These results suggest that CoA-flavonoids and resveratrol exert their beneficial effects through distinct but complementary pathways, influencing both gut microbiota composition and metabolic health. While the previous literature, particularly that on resveratrol, has documented some of these effects [56], the differences observed here, particularly in β-diversity and species-specific changes, underscore the need for further investigation of the specific roles of CoA-flavonoids in modulating the gut microbiota. This emerging field offers interesting potential for therapeutic strategies aimed at ameliorating metabolic disorders through targeted dietary interventions.

One aspect that is very variable in the consumption of bioactive compounds is the dosage; in the literature, there is a wide range of concentrations, depending on the expected effect. In the present study, a concentration of 0.02% of the diet was selected for all compounds, since this concentration led to an intake of approximately 75 mg/k/day. This concentration is in the range of the different metabolic effects previously reported with naringenin consumption, for which the average of the different doses reported in these studies included beneficial effects on obesity, the cardiovascular system and anti-inflammatory properties [18,48].

In recent years, there have been a few studies that have tried to improve the bioavailability of bioactive compounds with different methodologies, such as nano-capsules [57]. However, despite the fact that in some studies the serum concentrations of bioactive compounds such as fisetin are increased with these modifications, there is little evidence in the literature on the biological effects of these modifications. In the present study, different beneficial effects were observed with the consumption of CoA-flavonoids, but the absence of experimental groups consuming the unmodified compounds for purposes of comparison with the effects observed with CoA-flavonoids prevents us from determining whether these modifications enhance the biological effects of the natural compounds or whether their effects are selective. Another limitation, and an important perspective, for this study is the need to determine whether changes in solubility increased the bioavailability of CoA-flavonoids or whether CoA-flavonoids are metabolized primarily by intestinal bacteria. Possibly, this aspect could partly explain why resveratrol modified the gut microbiota differently, compared to CoA-flavonoids. More studies are required, but the evidence from this work showed that these compounds have an important biological activity.

It is important to highlight that some effects observed with the consumption of CoA-flavonoids, such as the improvement of hepatic lipid metabolism, the increases in energy expenditure and metabolic flexibility, and the boost in glucose metabolism, as well as the activation of adipose tissue thermogenesis, have been shown as possible alternatives when seeking to diminish conditions caused by metabolic diseases, and this indicates a comprehensive view of how CoA-flavonoids can control metabolic syndrome in its multiple aspects, such as lipid metabolism, inflammation and energy regulation, suggesting that co-amorphous compounds could play an important role in the treatment of diseases related to obesity, such as hepatic inflammation and abnormal lipid accumulation, as well as adipose tissue dysfunction. These findings suggest that these compounds could be an alternative used to prevent metabolic abnormalities in different tissues as a clinical treatment. However, further studies are needed to determine the possible long-term effects of CoA flavonoid consumption as well as its effect in humans.

## 5. Conclusions

This study provides evidence for the metabolic benefits of bioactive co-amorphous compounds such as CoA-naringenin and CoA-fisetin, especially in the context of obesity and metabolic syndrome. Importantly, this study demonstrated that CoA-flavonoids have distinct metabolic effects at different levels depending on the compound, indicating a differential and selective effect of each compound. Although the results for each CoA-flavonoid show that some metabolic aspects altered by HFSD consumption do not improve with its intake, the beneficial effects of the two compounds are different, and it would be interesting to explore whether a combination of both could improve their metabolic benefits and create a synergy between them.

## Figures and Tables

**Table 1:** Composition of the different diets, the control AIN-93 diet and the high-fat, high-sucrose diet (HFSD), with or without NPS-flavonoids and resveratrol.

Ingredients	Control (%)	HFSD (%)	HFSD NPS-Flavonoids/(%)	HFSD Resveratrol (%)
L-Cystine	0.3	0.3	0.3	0.3
Choline bitartrate	0.25	0.25	0.25	0.25
Vitamin mix	1	1	1	1
Fiber	5	5	5	5
Mineral mix	3.5	3.5	3.5	3.5
Soybean oil	7	3.1	3.1	3.1
Cornstarch	39.74	9	8.8	8.8
Dextrinized cornstarch	13.2	11.4	11.4	11.4
Sucrose	10	21.3	21.3	21.3
Casein	20	24	24	24
Lard	0	21.88	21.88	21.88
TBHQ	0.0014	0.0013	0.0013	0.0013
CoA-Flavonoids (Naringenin/fisetin)	--	--	0.2	--
Resveratrol	--	--	--	0.2
**TOTAL**	99.99	100.73	100.73	100.73

TBHQ: Tert-butylhydroquinone.

## Data Availability

The 16S rRNA gene sequencing raw sequence reads (fastq) are available at the NCBI Sequence Read Archive, with BioProject ID PRJNA1173341. The available data are accessible to the reviewers at the following link: https://dataview.ncbi.nlm.nih.gov/object/PRJNA1173341?reviewer=ou6la82706vdte1hr4ounsj2vu (accessed on 16 October 2024).
